# Environmental and Organismal Predictors of Intraspecific Variation in the Stoichiometry of a Neotropical Freshwater Fish

**DOI:** 10.1371/journal.pone.0032713

**Published:** 2012-03-06

**Authors:** Rana W. El-Sabaawi, Tyler J. Kohler, Eugenia Zandoná, Joseph Travis, Michael C. Marshall, Steven A. Thomas, David N. Reznick, Matthew Walsh, James F. Gilliam, Catherine Pringle, Alexander S. Flecker

**Affiliations:** 1 Department of Ecology and Evolutionary Biology, Cornell University, Ithaca, New York, United States of America; 2 School of Natural Resources, University of Nebraska, Lincoln, Nebraska, United States of America; 3 Department of Biology, Drexel University, Philadelphia, Pennsylvania, United States of America; 4 Department of Biological Science, Florida State University, Tallahassee, Florida, United States of America; 5 Odum School of Ecology, University of Georgia, Athens, Georgia, United States of America; 6 Department of Biology, University of California Riverside, Riverside, California, United States of America; 7 Department of Ecology and Evolutionary Biology, Yale University, New Haven, Connecticut, United States of America; 8 Department of Biology, North Carolina State University, Raleigh, North Carolina, United States of America; Biodiversity Insitute of Ontario - University of Guelph, Canada

## Abstract

The elemental composition of animals, or their organismal stoichiometry, is thought to constrain their contribution to nutrient recycling, their interactions with other animals, and their demographic rates. Factors that affect organismal stoichiometry are generally poorly understood, but likely reflect elemental investments in morphological features and life history traits, acting in concert with the environmental availability of elements. We assessed the relative contribution of organismal traits and environmental variability to the stoichiometry of an insectivorous Neotropical stream fish, *Rivulus hartii*. We characterized the influence of body size, life history phenotype, stage of maturity, and environmental variability on organismal stoichiometry in 6 streams that differ in a broad suite of environmental variables. The elemental composition of *R. hartii* was variable, and overlapped with the wide range of elemental composition documented across freshwater fish taxa. Average %P composition was ∼3.2%(±0.6), average %N∼10.7%(±0.9), and average %C∼41.7%(±3.1). Streams were the strongest predictor of organismal stoichiometry, and explained up to 18% of the overall variance. This effect appeared to be largely explained by variability in quality of basal resources such as epilithon N∶P and benthic organic matter C∶N, along with variability in invertebrate standing stocks, an important food source for *R. hartii*. Organismal traits were weak predictors of organismal stoichiometry in this species, explaining when combined up to 7% of the overall variance in stoichiometry. Body size was significantly and positively correlated with %P, and negatively with N∶P, and C∶P, and life history phenotype was significantly correlated with %C, %P, C∶P and C∶N. Our study suggests that spatial variability in elemental availability is more strongly correlated with organismal stoichiometry than organismal traits, and suggests that the stoichiometry of carnivores may not be completely buffered from environmental variability. We discuss the relevance of these findings to ecological stoichiometry theory.

## Introduction

Ecological stoichiometry expresses ecological interactions and biogeochemical processes as proportions of essential elements (C, N and P), whose flux is governed by mass-balance relationships. A fundamental concept in ecological stoichiometry is that animals are largely homeostatic, meaning that they regulate and maintain their elemental composition in the face of variable elemental composition in their diets [1]. Mismatches between the elemental requirements of an animal and the supply of elements in its diet constrain growth and reproduction, which can have subsequent effects on a range of ecological, biogeochemical and physiological processes [2], [3]. In this context the elemental composition of organisms, or their organismal stoichiometry, act as a proxy for their nutritional demand, and can constrain their ecosystem function, behavior and community interactions [4]–[7].

Organismal stoichiometry is thought to reflect macromolecular composition. For example, P content reflects P-rich molecules such as RNA, DNA and apatite [8], [9], while C content is correlated with lipids or carbohydrates, and nitrogen content with protein [1], [10]. Patterns of investments in organismal traits such as growth rate, reproduction, body size or morphology can thus be strongly correlated with patterns of organismal stoichiometry [8], [11], [12]. Increasing RNA content during growth, for example, results in a significant positive correlation between P content and growth rate in invertebrates and microorganisms [8]. Less is known about the stoichiometric costs of life history traits in other taxa, but growth rate has been shown to correlate with the N∶P ratio of freshwater fish, with slow-growing fish displaying lower N∶P ratios that fast growing fish, presumably because fast-growing fish incorporate proportionally more muscle relative to bone [13], [14]. Reproductive status may also influence %C and C∶N in fish likely because reproduction affects lipid accumulation in somatic and reproductive tissues [10]. Investments in skeletal support as fish grow larger produce a positive relationship between P content and body size in many species [9].

However, other studies have shown that resource availability and quality can also significantly influence organismal stoichiometry. Correlations between the elemental content of organisms and the elemental content of their diets, or the elemental content of basal resources in their systems, have been demonstrated in a broad range of herbivorous and detritivorous taxa, from terrestrial and aquatic systems [15]–[17]. For example, whole-stream nutrient enrichment can increase nutrient content in almost all basal resources, and in some invertebrate taxa that rely on these resources [17]. Similar enrichments have been shown to affect the stoichiometry of omnivorous and carnivorous invertebrates suggesting that variability in basal resource quality can affect multiple trophic levels, not just primary consumers [18]. In a recent experiment, the stoichiometry of an omnivorous fish was shown to vary in response to the quantity and quality of phytoplankton, which are modulated by nutrients and light [19]. It is thus possible that variation in organismal stoichiometry is more likely to reflect variability in environmental conditions that influence elemental availability in dietary or basal resources than variation in organismal traits. This implies that animals are more flexible and less homeostatic in their elemental composition than originally thought. Variability in the strength of elemental homeostasis has consequences for fitness and competitive ability [20].

Few studies have attempted to simultaneously assess the relative contribution of these two potential sources of variability to organismal stoichiometry within a single species. As a result, we do not know the relative strength of each in the face of the other. Of the few that have made this attempt, nearly all have focused on invertebrates. For example, Bertram et al. [12] found that almost half of the variability in organismal stoichiometry of terrestrial insects was attributed to environmental heterogeneity, while the other half was attributed to variability in body size. In contrast, very little is known about the relative influence of organismal traits and environmental heterogeneity on stoichiometry in fish, even though those taxa are often the most important nutrient recyclers in streams and lakes, and are typically the largest pools of N and P in those systems [21], [22]. Most studies on intraspecific stoichiometry in fish have focused on the contribution of organismal traits such as ontogeny or body size to organismal stoichiometry [9], but recent evidence suggests that environmental and dietary factors may also play a role [8], [19], [23].

Here we assess how organismal traits influence organismal stoichiometry of an insectivorous fish across a range of environmental conditions. *Rivulus hartii* is a strictly insectivorous stream fish that is widely distributed in northern South America and the southern Caribbean, where it is often the lone species in a stream, and where it can reach high densities [24]. In Trinidad, where our study took place, *R. hartii* is distributed across sites that differ widely in nutrient loading, hydrological conditions and light availability, all of which have been shown to influence the quantity and quality of basal resources in this system [25], [26]. In these streams *Rivulus hartii* also displays three distinctive life history phenotypes that represent tradeoffs in growth and reproduction [27]. This system allowed us a unique opportunity to simultaneously assess how trait variation related to life history, body size or stage of maturity might influence organismal stoichiometry across a wide range of environmental conditions that modulate the quality and availability of basal resources. We hypothesize that body size will be positively correlated with %P because larger fish typically contain more P-rich bone than smaller fish [9]. We also hypothesize that fish %C and N∶P will be correlated with life history phenotype because life history phenotype influences reproduction and growth in this species, and because reproduction and growth are correlated with organismal stoichiometry in other fish species [13], [28]. We also predict that organismal stoichiometry of *R. hartii* will vary in response to environmental heterogeneity that creates differences in the quality and quantity of stream basal resources. For example, stoichiometric ratios of *R. hartii* will be correlated with the stoichiometric ratios of basal resources, or with environmental factors such as light and nutrient loading that influence the ratios of basal resources.

## Methods

### Site choice and characteristics

Trinidadian streams are characterized by barrier waterfalls that restrict the migration of fish, creating distinct longitudinal fish communities characterized by different biotic interactions. In low-elevation streams fish communities are most diverse, containing piscivores such as *Hoplias malabaricus* and *Crenicichla frenata* or *C. saxatilus*. Fish species richness declines with increasing elevation due to natural barriers. Fish species composition of mid-elevation streams is often limited to *R. hartii* and guppies (*Poecilia reticulata*), while *R. hartii* is often the only fish species in high elevation sites [24], [29]. The life history phenotype of *R. hartii* is determined by the composition of the fish community. At sites where *R. hartii* are found with predators (High Predation, HP sites), they mature at a relatively young age, produce many small eggs, and are generally dominated by small, fast growing individuals [24], [27], [29]. At sites where they are the only fish species (*R. hartii* only, RO sites), *R. hartii* mature late in life, produce few large eggs per clutch, and are dominated by large, slow growing individuals [27], [30]. At sites where they are found with guppies (*R. hartii*-guppy site, RG), *R. hartii* display a life history phenotype that is intermediate between HP and RO locations [27], [31]. Differences between these phenotypes have been shown to be genetic and heritable, and are also inducible by transplanting guppies from HP systems to RO sites upstream of a barrier waterfall [27], [30], [32]. Based on these differences in life history we would expect both C∶N and N∶P to decline progressively from RO to HP locations.

**Figure 1:**
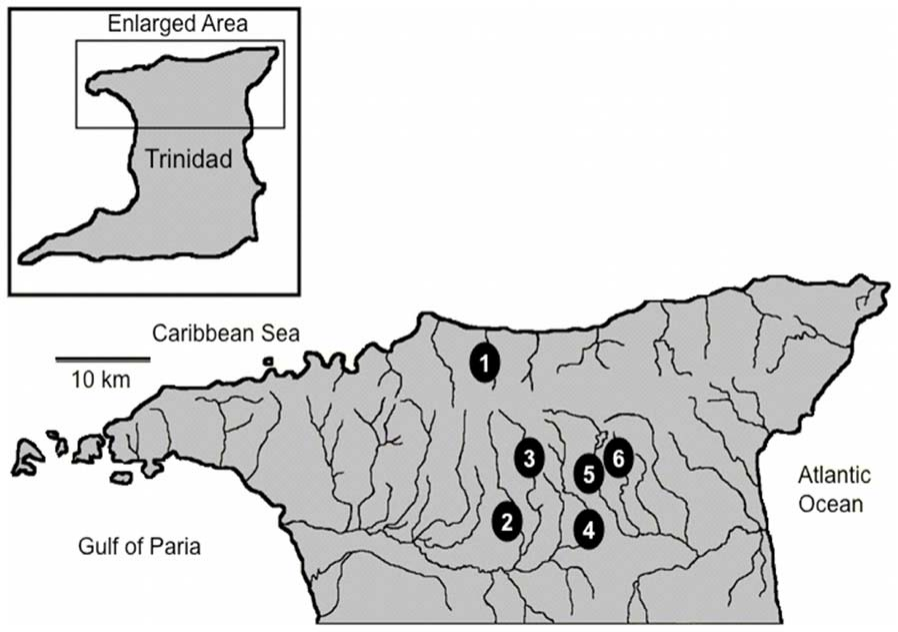
Map displaying locations sampled in this study. (1) Marianne, (2) Arima, (3) Guanapo, (4) Aripo, (5) Quare, and (6) Turure. All streams were located in the Northern Range Mountains of Trinidad and Tobago (location shown in inset).

We sampled *Rivulus hartii* from 18 populations distributed in 6 streams in the Northern Range of Trinidad (Fig. 1). These streams included the Aripo, Arima and Guanapo Rivers (from the Caroni Drainage located on the South Slope of the range), the Marianne (from the Marianne Drainage located in the North Slope), and the Turure and Quare (both from the Oropouche Drainage located in the East Slope) Rivers. In each stream, we sampled three sites representing the three fish communities: *R. hartii* only (RO), *R. hartii* and guppy only (RG) and *R. hartii*, guppy and predators (HP). Fish life history phenotype had been previously characterized from all of these streams [27], [33]. Fish community composition was found to be the strongest predictor of life history characteristics, suggesting that the environmental component of life history phenotype was not as strong as the genetic component [27].

These sites also displayed a wide range of environmental variability that spanned an order of magnitude for nutrient concentration, light availability (measured as % open canopy) and stream discharge (Table 1, sample methods reported in Appendix S1). These factors can influence the quality and availability of basal resources in these streams and other systems [17], [19], [25], [26]. There was considerable variability in the elemental content and stoichiometry of two basal resources, epilithon and benthic organic matter (Appendix S1 and Table S1). Invertebrate standing stocks also varied substantially (Table 1), and those are an important source of food for this species [34]. In some cases, sites within a stream were likely to have similar environmental conditions. For example, average dissolved inorganic nitrogen was lower in the Marianne than in other rivers, while averaged dissolved phosphorus was lower in the Quare and Turure than other rivers (Table 1). In other variables, such as algal or invertebrate standing stocks, variability within stream appeared to be as large as between streams (Table 1). There were no significant differences in temperature, dissolved oxygen content or pH between sites (see Appendix S1).

**Table 1:** Environmental characteristics among *Rivulus hartii* sites.

Stream	Community	Discharge	Benthic organic matter	Invertebrate standing stocks	Light	Algal standing stocks	Epilithon standing stocks	DIN	Phosphorus	Ammonium
		(L/S)	(g dry mass/m2)	(mg/m2)	(% open canopy)	(mg Chl a/m2)	(g AFDM/m2)	(µg N/L)	(µg P/L)	(µg N/L)
Arima	HP	32	9.9(8.9)	972(861)	33(20)	5.2(5.6)	2.2(2.1)	899(101)	70(13)	2(0.5)
	RG	15.8	17.2(15.7)	209(266)	10(7)	4.8(7.8)	3.98(2.8)	398(7)	16(8)	1.1(0.5)
	RO	25	17.6(17.7)	144(99)	6(6)	14.1(11.8)	4.8(4.0)	319(61)	3(1)	2.7(0.5)
Aripo	HP	52.7	15.3(15.0)	5517(1866)	25(10)	8.4(11.5)	3.1(1.6)	184(37)	26(11)	6.8(1.3)
	RG	41.1	57.0(83.3)	700(628)	7(6)	11.0(15.3)	7.6(4.0)	500(192)	6(3)	5.3(2.7)
	RO	2	14.6(19.9)	396(566)	6(3)	5.4(8.1)	9.7(8.0)	104(53)	9(4)	3.2(2.5)
Guanapo	HP	N/A	10.0(13.0)	1399(754)	18(10)	4.5(7.1)	2.4(1.2)	195(81)	37	2.4(1.9)
	RG	32.6	21.7(22.3)	2431(1799)	11(10)	5.0(12.6)	11.1(11.0)	208(39)	36(11)	3.1(0.4)
	RO	N/A	44.9(45.7)	927(858)	13(6)	7.3(16.9)	7.4(6.0)	324(62)	25(1)	2.6(1.3)
Marianne	HP	1323.3	16.6(16.5)	2164(1306)	19(11)	5.4(11.6)	3.0(1.9)	110(78)	13(2)	5.0(2.4)
	RG	147.6	19.1(22.5)	816(476)	11(7)	6.0(10.3)	3.7(3.4)	74(28)	5(2)	2.0(0.2)
	RO	N/A	47.5(67.6)	547(270)	8(5)	5.9(9.5)	3.2(1.8)	82(27)	4(3)	3.6(3.3)
Quare	HP	57.5	8.7(9.8)	415(233)	47(17)	15.2(17.1)	4.1(2.7)	187(9)	5(2)	2.7(1.3)
	RG	11.9	10.1(9.8)	38(47)	8(6)	4.0(6.7)	5.3(4.5)	75(11)	10(6)	1.2(0.3)
	RO	55.7	24.4(20.9)	73(47)	5(3)	6.1(6.8)	5.9(4.3)	512(2)	5(3)	1.6(0.1)
Turure	HP	157.4	10.3(8.6)	89.1(151)	22.2	12.1(10.8)	3.1(2.7)	137(86)	6(3)	3.0(0.2)
	RG	N/A	N/A	N/A	14.9	39.7(57.2)	16.9(11.9)	477(5)	5(2)	1.6(0.0)
	RO	60.1	10.8(9.0)	28.1(15)	11.9	8.5(4.8)	3.3(2.1)	202(23)	11(3)	1.8(0.1)

HP indicates high predation sites, RG indicates *R. hartii*/guppy sites, while RO indicates *R. hartii* only sites. Values are means and brackets represent standard deviations of three replicates when available (see methods). N/A indicates sites where logistical difficulties hindered collection of environmental variables. Sampling of environmental variables is described in Appendix S1.

### Fish stoichiometry

Sampling occurred in July 2007 and July 2008, at the transition between dry and wet season conditions. At each site individual *R. hartii* were collected with dip nets from three representative pools. A total of 240 fish were collected; at most sites, we were able to catch 10–25 individuals, except for the Aripo HP and Guanapo RO, where we were only able to collect 6 and 9 fish, respectively. Body size of *R. hartii* varied between 10 and 84 mm, but maximum collected body size was slightly lower in HP locations (61 mm) compared to RG and RO locations (84 and 76 mm respectively). The sampling was biased by stage of maturity (70% adults, 30% juveniles), but not by community composition (approximately a third of the sample for each community type). Juveniles were particularly difficult to find in the Aripo, Guanapo and Marianne rivers. Animal handling was approved by The University of Georgia's Institutional Animal Care and Use Committee Protocol (A2007-10107-0, Michael Marshall and Catherine Pringle PIs). Fish collection and export was approved by Ministry of Agriculture, Land and Marine Resources, Republic of Trinidad and Tobago.

Fish were placed immediately on ice after collection. In the laboratory they were classified as adults or juveniles based on the presence of reproductive tissues and on coloration of the tail. Guts were removed, and fish were dried at 50°C until a constant dry mass was achieved, and were then ground into a fine powder using a mortar and pestle. Subsamples were analyzed for %C and %N content using a Carlo Erba NA1500 CHN analyzer. Subsamples for particulate P analysis were ashed at 500°C, and then digested with HCl at 102°C for two hours. The concentration of dissolved P from the digested solution measured using the molybdate method [35]. Bone meal was used as a standard for the method. Three replicates of the same sample were run whenever possible. Hereafter elemental composition refers to the elemental percent of dry mass (%C, %N and %P), while stoichiometry refers to the molar ratios (C∶N∶P) of these elements.

### Statistical analysis

A general linear model (GLM) was used to assess the relative contributions of various predictive factors to the elemental composition and stoichiometry of *R. hartii*. The predictors included the life history phenotype determined by fish community composition (RO, RG and HP), stage of maturity (adults and juveniles), and body size (modeled as a covariate). The environmental component of organismal stoichiometry was modeled using stream as a predictive factor or as the interaction of stream×community composition. A significant stream effect would indicate a consistent influence of the stream of origin, a broad surrogate for environmental effects of all sorts. A significant main effect of community would indicate consistent differences associated with the repeatable differences in life history phenotype found in different communities. A significant stream×community interaction would indicate that the effect of community on organismal stoichiometry differed by stream, either because the community effect might be stronger in some streams than others, or because there is extensive local variation among the sampled sites that is not predictable in any consistent fashion by the specific community composition and individual stream.

All variables were modeled as fixed effects, including stream because we were interested in assessing the contribution of different streams to elemental composition and stoichiometry, and because we specifically chose streams that are well-studied and where life history phenotype of *R. hartii* has been characterized [36]. Initial model runs included the interactions of body size×stream and community×body size but those were removed because they were insignificant (P>0.05), and because their F-ratio was less than 1. Because the distribution of juveniles was skewed by stream, we ran two different sets of GLMs. The first set was constrained to adult fish, which were evenly distributed across all streams and communities. The second set was used to assess the effect of the stage of maturity on organismal stoichiometry in those streams where juvenile sample numbers were evenly distributed across all community types (i.e. the Arima, Quare and Turure). This approach minimized any statistical bias that might have resulted from the uneven distribution of samples across streams or the undue influence of small sample sizes in distinct subsets of treatment combinations.

Separate models were constructed for %P, %C, %N, C∶P, C∶N and N∶P. The proportion of the variance explained by each predictor, or its effect size, was computed using eta squared (η^2^), which is the sum of squares of each variable divided by the sum of squares of the overall model. All data were tested for normality and heterogeneity of variance before analysis, and were transformed if necessary.

When either stream or stream×community were found to be significant predictors of organismal stoichiometry, we assessed whether this pattern was driven by a particular environmental variable (Table 1 and Table S1). We did this by first correlating least squares means of stoichiometric ratio at each site (generated by the previously-described GLM) against a subset of environmental variables reported in Table 1 and Table S1. These included invertebrate standing stocks, algal standing stocks, light, dissolved ammonium, dissolved phosphorus. Discharge was not included in the analysis because it did not have enough data points for a robust analysis. In addition, each stoichiometric ratio was also correlated against the corresponding stoichiometric ratios of either epilithon or benthic organic matter. As such, each stoichiometric variable was correlated against 10 environmental variables. The strength of these correlations was assessed using Pearson's correlation coefficient (r at α = 0.05, which was corrected for multiple comparisons).

When a significant correlation between an environmental variable and least squares mean organismal stoichiometry was discovered, we assessed how much of the stream effect was explained by that environmental variable. To do so, we compared the sum of squares generated from a GLM that included only the stream effect, a GLM that included only the environmental variable, and a GLM that include both the stream and the environmental variable. Models that include a single predictor evaluate the individual contribution of that predictor to the overall variance (Type 1 sum of squares), while models that contain two predictors evaluate the contribution of each one when the effect of other was accounted for (Type 3 sum of squares). If both variables explained overlapping proportions of the overall variance, we would expect the sum of squares of each variable from models that contains both variables to be smaller than the sum of squares from models that include only one predictor. All statistical analysis was conducted using JMP software (Version 8 for Macintosh computers).

## Results

### General patterns in the organismal stoichiometry of *R. hartii*

*Rivulus hartii* displayed substantial intraspecific variability in its elemental composition and stoichiometry. Among individuals, % P was the most variable of the elements (coefficient of variation (CV∼19.8%), followed by nitrogen (CV∼8.4%) and then carbon (CV∼6.7%) (Table 2). Average %P composition was ∼3.2%(±0.6), average %N composition was ∼10.7%(±0.9), and average %C composition was ∼41.7%(±3.1). Of the stoichiometric ratios C∶P was the most variable (CV∼23%), followed by N∶P (CV∼19.9%) with C∶N having the least variation (CV∼8.9%) (Table 2).

**Table 2:** The distribution of elemental composition in all *Rivulus hartii* individuals collected in this study compared to the distribution of elements and elemental ratios of 31 fish species compiled in a recent review [14].

Data source	Variable	Mean	Median	Std Dev	Min	Max	Coefficient of Variability
All individuals	%P	3.2	3.2	0.6	1.8	5	19.8
(n = 240)	%N	10.7	10.8	0.9	8.6	13.2	8.4
	%C	41.7	41.5	3.2	30	51.8	7.6
	C/P	35.4	33.4	10.4	18.7	79.8	22.8
	N/P	7.8	7.5	2	4.6	12.7	19.6
	C/N	4.6	4.4	0.4	3.8	6.3	9.5
Fish species	%P	2.9	N/R	N/R	1.3	5.7	29.8
	%N	10.1	N/R	N/R	6.7	13.2	12
	%C	44.7	N/R	N/R	30.2	53.6	8.3
	C/P	44	N/R	N/R	15.9	95.9	33
	N/P	8.4	N/R	N/R	2.8	16.1	30
	C/N	5.2	N/R	N/R	3.8	7.7	13.9

N/R indicates that values were not reported in the original study.

### Correlates of organismal stoichiometry in adult *R. hartii*

**Figure 2:**
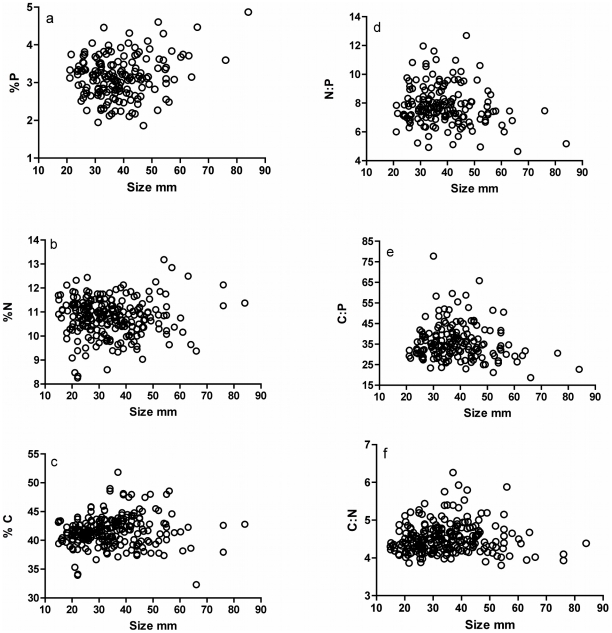
Correlations between body size, elemental composition or organismal stoichiometry of adult *Rivulus hartii*.

Adult body size was significantly and positively associated with %P (Table 3, Fig. 2) but displayed no relationship with %C or %N. Consequently, larger adults had significantly reduced ratios of N∶P and C∶P (Fig. 2). However, much of this relationship was influenced by the presence of a few large individuals, and removing those individuals from the analysis weakened correlations between %P, N∶P, C∶P and body size, and reduced its η^2^ from 0.03–0.04 to ∼0.01 (P<0.05) (Fig. 2).

**Table 3:** Analysis of elemental composition and stoichiometry of adult *Rivulus hartii* using a general linear model (GLM) and variance decomposition (η^2^).

GLM	Source	DF	%P	%N	%C	N∶P	C∶P	C∶N
	Stream	5.00	7.67**	6.33**	4.42**	5.35**	6.39**	7.41**
	Community	2.00	2.7* #	0.03	5.51**	1.31	3.51*	3.60*
	Size	1.00	8.67**	0.01	1.32	5.53**	7.97**	0.41
	Stream×Community	10.00	1.41	2.33**	1.04	0.84	1.05	2.23*
	r^2^		0.32	0.28	0.27	0.21	0.28	0.33
								
η^2^	Stream		0.18	0.15	0.11	0.14	0.16	0.17
	Community		0.03	<0.01	0.05	0.01	0.03	0.03
	Size		0.04	<0.01	0.01	0.03	0.04	<0.01
	Stream×Community		0.07	0.11	0.05	0.04	0.05	0.10

All values are F ratios, and symbols indicate degree of significance. DF is degrees of freedom used for each variable. C∶P, N∶P and C∶N were log transformed before analysis.

** indicates P<0.01.

* indicates P<0.05.

# P∼0.043.

**Figure 3:**
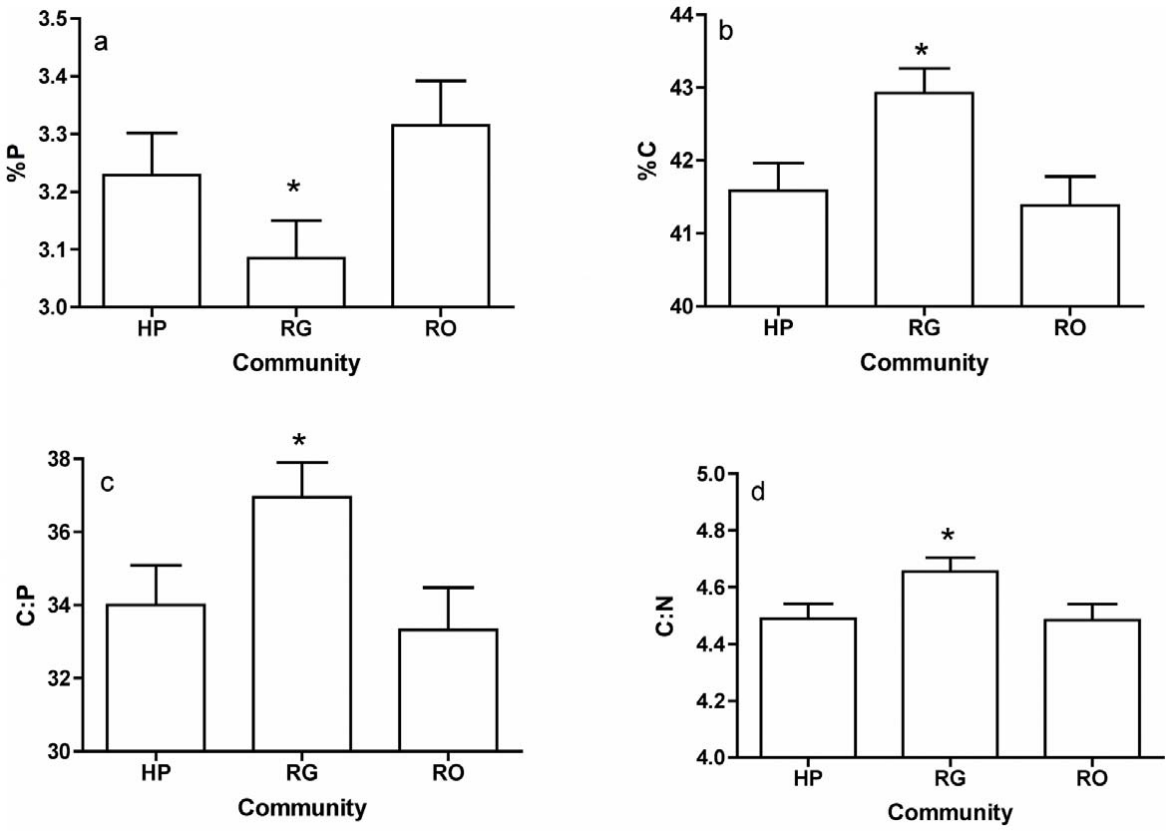
The effect of community composition (i.e. life history phenotype) on the elemental stoichiometry of adult *Rivulus hartii*. Fish community composition predicts life history phenotype of this species, as well as a small component of its elemental composition and organismal stoichiometry. Bars are least squares means (standard error) of elemental composition and elemental stoichiometry generated from a general linear model (Table 3). They are standardized to body size = 35 mm. Community designations are RO = *Rivulus* Only, RG = *R. hartii* and Guppies, HP = High Predation sites. Bars surmounted by a star are significantly different using Tukey post hoc HSD test at P<0.05.

Several stoichiometric variables varied significantly with fish community, and consequently, life history phenotype. Adults from sites with guppies (RG) displayed lower %P, higher %C, C∶N and C∶P than adults from populations where *R. hartii* occurred alone (RO) or with other predators (HP) (Table 3, Fig. 3). Nevertheless, these significant effects were relatively weak, whether considered in terms of the percent differences between average values found in RG adults and those from the other communities (Fig. 3), or in terms of P values (∼0.04), or in terms of the statistical strength of the effect as measured by η^2^ (Table 3).

**Figure 4:**
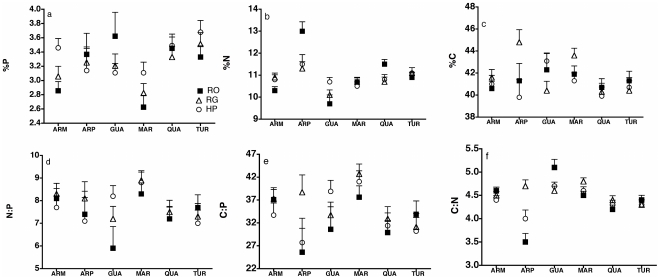
Spatial variability in the organismal stoichiometry of adult *Rivulus hartii*. Stream and the interaction of stream×community are the strongest predictors of elemental composition and organismal stoichiometry of *R. hartii*. Values are least squares means generated from a general linear model (Table 3). They are standardized to body size = 35 mm. Community designations are RO = *Rivulus* Only, RG = *R. hartii* and Guppies, HP = High Predation sites. ARM is the Arima, ARP is Aripo, GUA is Guanapo, MAR is Marianne, QUA is Quare and TUR is the Turure.

The strongest predictor of C, N, and P elemental composition and stoichiometric ratios was the stream where samples were collected (Table 3, Fig. 4). Adult *R. hartii* from different streams were likely to have significantly different elemental content or stoichiometric ratios (Fig. 4). Furthermore, there was a strong interaction between stream and fish community in %N and the C∶N ratio. This indicated that variability in %N and C∶N was explained mostly by variability in local site conditions, rather than variability in community composition (Fig. 4). This effect was primarily driven by fish from the Guanapo, Aripo, and to a lesser extent, the Quare and the Arima, where fish from different individual locations in the same stream had significantly different C∶N composition (Fig. 4).

### Comparing juvenile and adult organismal stoichiometry in *R. hartii*

There were no consistent differences between adults and juveniles from the Arima, Quare and Turure rivers in elemental composition or stoichiometric ratios (Table 4). However, we observed a strong community×stage of maturity interaction in %C, C∶N and C∶P indicating that the association between fish community composition and organismal stoichiometry was only significant in adult *R. hartii* (Table S2). In contrast, the association between %P and community was similar in both adults and juveniles (Table 3 and Table 4).

**Table 4:** Analysis of elemental composition and stoichiometry of *Rivulus hartii* using a general linear model (GLM) and variance decomposition (η^2^).

			%P	%N	%C	N∶P	C∶P	C∶N
GLM	Stream	2	5.34**	10.56**	0.69	1.18	3.95**	13.35**
	Community	2	3.13*	2.66	2.66#	3.35	3.59*	2.06
	Size	1	3.29#	2.1	1.09	2.64#	2.57#	0.51
	Stage of maturity	1	1.59	0.27	0.49	0.49	0.77	0.5804
	Community×Stage of maturity	2	1.82	1.4	10.01**	2.49	4.06*	8.89**
	Stream×Community	4	0.98	2.98**	0.99	0.33	1.04	4.19**
		r^2^	0.17	0.27	0.18	0.13	0.19	0.35
η^2^	Stream		0.07	0.13	0.01	0.02	0.05	0.14
	Community		0.04	0.03	0.04	0.05	0.05	0.02
	Size		0.02	0.01	0.01	0.02	0.02	<0.01
	Stage of Maturity		0.01	<0.01	<0.01	<0.01	0.01	<0.01
	Community×Stage of maturity		0.02	0.02	0.13	0.04	0.05	0.10
	Stream×Community		0.03	0.07	0.03	0.01	0.03	0.09

This analysis was restricted to populations from the Arima, Quare and Turure rivers, where the number of juveniles was evenly distributed across all sites. All values are F ratios, and symbols indicate degree of significance. DF is degrees of freedom used for each variable. C∶P, N∶P and C∶N were log transformed before analysis.

** indicates P<0.001,

* indicates P<0.05,

# indicates P between 0.05 and 0.07.

Trends observed in models that included adults and ones that included adults and juveniles were largely in agreement, with a few minor exceptions. Stream was not a significant predictor of N∶P and %C in the model that included adults and juveniles, likely because this model was limited to Arima, Quare and Turure, which were not dramatically different in their N∶P or %C (Fig. 4). The relationships between size and %P, N∶P and C∶P were similar but weaker in the model that included juveniles compared to the model that was restricted to adults (Table 3 and Table 4).

### Correlations between organismal stoichiometry and individual environmental variables

**Figure 5:**
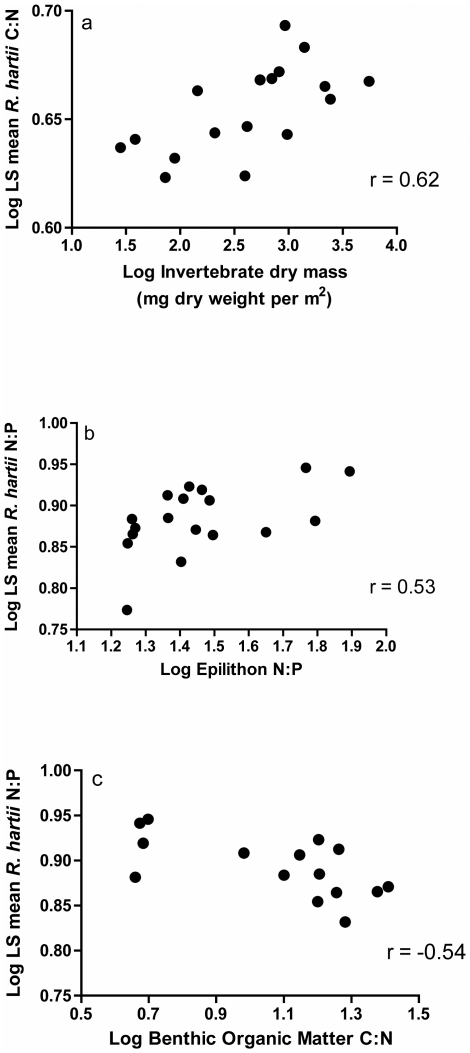
Correlations between resources and organismal stoichiometry of adult *Rivulus hartii*. Different aspects of the organismal stoichiometry of adult *R. hartii* are significantly correlated with the invertebrate standing stocks (a), with the stoichiometry of epilithon (b), and with the stoichiometry of benthic organic matter (c).

The C∶N ratio of adult *R. hartii* was significantly correlated with invertebrate standing stocks (r = 0.62, P<0.001) (Fig. 5a), whereas the N∶P ratio of adult *R. hartii* was significantly correlated with the N∶P of epilithon (r = 0.53, P = 0.004)(Fig. 5b). The N∶P ratio of adult *R. hartii* was also significantly and negatively correlated with C∶N of benthic organic matter (ρ = −0.54, P = 0.002). We were not able to attribute these patterns to variability in a single element (i.e. %N, %C or %P) in *R. hartii*. In addition, invertebrate standing stocks were significantly confounded with two other variables: epilithon C∶N and benthic organic matter C∶N. However, invertebrate standing stocks were a much stronger predictor of *R. hartii* C∶N than benthic organic matter C∶N or epilithon C∶N, and residuals from the correlation between *R. hartii* C∶N and invertebrate standing stocks were not significantly correlated with either benthic organic matter C∶N, or with epilithon C∶N (analyses not shown). In addition, invertebrate standing stocks, epilithon N∶P and benthic organic matter C∶N explained a significant proportion, but not all, of the effect of stream on organismal stoichiometry (Table 5). The sum of squares of each variable was considerably smaller in models that contained both predictors than models that contained single predictors (Table 5). In all cases the influence of streams on organismal stoichiometry was stronger than the influence of epilithon quality, invertebrate standing stocks or benthic organic matter quality. The C∶P ratio of *R. hartii* was not correlated with any environmental variable measured in this study (P>0.005).

**Table 5:** A statistical assessment of how much of the stream effect in organismal stoichiometry is described by the quality of benthic resources or by the overall availability of resources.

Model×variable(s)	Model Y variable	Adjusted r2	P	Overall SS	SS for individual variables
Epilithon N∶P*	*R. hartii* N∶P	0.07	<0.001	25.6	
Stream	*R. hartii* N∶P	0.15	<0.001	56.8	
Stream, Epilithon N∶P*	*R. hartii* N∶P	0.15	<0.001	56.86	SS of Stream = 15.75
					SS of Epilithon N∶P = 0.08
Benthic organic matter C∶N*	*R. hartii* N∶P	0.11	<0.001	32.9	
Stream	*R. hartii* N∶P	0.15	<0.001	56.8	
Stream, benthic organic matter C∶N*	*R. hartii* N∶P	0.17		40.3	SS of Stream = 21.50
					SS of benthic organic matter C∶N = 1.6
Invertebrate standing stocks*	*R. hartii* C∶N	0.05	0.003	1.4	
Stream	*R. hartii* C∶N	0.16	<0.001	4.81	
Stream, invertebrate standing stocks*	*R. hartii* C∶N	0.14	0.006	3.85	SS of stream = 2.4
					SS of standing stocks = 0.19

* values were log transformed prior to analysis.

## Discussion

Intraspecific stoichiometry in *R. hartii* is widely variable, and spans a considerable range of stoichiometry observed across freshwater fish taxa [14]. Earlier studies described intraspecific variation in fish stoichiometry as narrow [3], but it is unclear whether sampling bias has influenced these findings. Some have suggested that most studies involving fish stoichiometry typically rely on a narrow range of individuals and thus may underestimate the range of elemental variability generated by factors such ontogenetic development or body size [9]. Broad intraspecific variability has been reported in many taxa including algae, plants, terrestrial and freshwater insects, and fish [12], [18], [37]. It is thus the norm, rather than the exception that intraspecific variability in organismal stoichiometry is wide. The ecological and physiological consequences of this variability are poorly understood, though they may be significant and complex. For example, intraspecific variability in organismal stoichiometry that is related to ontogenetic development can create bottlenecks in population growth rates [38], but may not necessarily influence ecosystem function [9]. Understanding the ecological significance of wide intraspecific variation in organismal stoichiometry in fish is especially important because they often represent the largest pools of N and P in freshwater systems and because they often have significant ecological roles in these systems [21].

### Environmental predictors of organismal stoichiometry of *R. hartii*

The strongest predictor of organismal stoichiometry in *R. hartii* is either the stream, or to a lesser extent, the interaction of stream×community. The former suggests that *R. hartii* from different rivers are likely to vary in their elemental composition. The latter suggests that local site conditions significantly modify %N and C∶N, and that *R. hartii* from different sites or communities in the same stream have the potential to be as variable as when compared across streams (Fig. 4). Spatial differences in organismal stoichiometry have been noted in other species of fish. In Bluegill (*Lepomis macrochirus*) % P is significantly correlated with lake identity [23], whereas in Eurasian perch (*Perca fluviatilis*) %P is strongly correlated with microhabitat use [11]. However, in both of those studies elemental composition was either confounded with strong differences in body morphology [11] or potential differences in life history traits [23] that may influence the stoichiometry of fish, and that may have caused the correlation between sampling location and elemental composition. Our findings suggest that sampling location is more important in explaining elemental composition than either stage of maturity, body size or fish community composition, which selects for life history phenotype in *R. hartii*.

Why are *R. hartii* from different streams significantly different in their organismal stoichiometry? The sites we chose for our study vary significantly in a wide suite of environmental variables, including the availability of food sources and the quality of basal resources (Table 1, Table S1). Assessing how organismal stoichiometry correlates with these variables is the first step towards generating hypotheses about factors that constrain organismal stoichiometry in nature. We found that *R. hartii* C∶N was positively correlated to invertebrate standing stocks (Fig. 5). Lipid content, for which C∶N is a proxy, has been shown to increase with food concentration in a number of fish species [39]. Aquatic invertebrates are an important diet for *R. hartii* [34]. The positive correlation between invertebrate standing stocks and *R. hartii* C∶N thus suggests that C∶N is affected by the availability of diet, and that *R. hartii* acquires more lipids when invertebrate standing stocks are high. It is striking that such a large variation in invertebrate standing stocks is correlated with a relatively small shift in C∶N, but studies have shown that comparable variability in C∶N may indicate up to a 15-fold shift in lipid content [10], [40]. Interestingly, invertebrate biomass was not significant correlated with *R. hartii* %C even though this element is abundant in lipids. The correlations between lipid content, C∶N and diet availability need to be specifically examined in feeding experiments.

We additionally found significant correlations between the N∶P ratio of adult *R. hartii*, and the stoichiometry of two important basal resources: epilithon and benthic organic matter (Fig. 5). There are two possible mechanisms that could explain this correlation. First, because animals can play a significant role in nutrient recycling, they can alter the concentrations of dissolved nutrients and affect the stoichiometry of basal resources, and this can lead to correlations between the stoichiometry of animals and the stoichiometry of basal resources [41]. However, even though *R. hartii* can be abundant, especially in RO sites, there are other animals in these streams whose excretion can potentially also influence dissolved nutrient concentrations [42]. In RG or HP sites, for example, *R. hartii* are a smaller proportion of the overall fish standing stocks, and their contribution to nutrient recycling in these sites is likely smaller than other fish, especially guppies, which have significantly higher nutrient recycling rates than *R. hartii* [43]. As such, it is unlikely that the correlations between *R. hartii* stoichiometry and basal resource stoichiometry are caused by *R. hartii*-mediated nutrient recycling.

An alternative explanation is that variation in the quality of basal resources constrains the elemental content of higher trophic levels. This has been experimentally demonstrated in carnivorous and omnivorous invertebrates [18] and in an omnivorous fish [19]. Epilithon stoichiometry and quality in Trinidadian streams are controlled by complex interactions between light availability, nutrient loading and discharge [25], [26]. Factors that influence the quality of benthic organic matter in these streams have not been formally explored, but are also likely to vary with nutrient loading and discharge [18]. Organismal stoichiometry is not significantly correlated with light, nutrient loading or stream discharge, likely because it is their interaction, rather than their individual effects, that affect organismal stoichiometry in these streams. The correlation between the stoichiometry of basal resources and animals in higher trophic levels suggests that the local or watershed scale drivers of stoichiometric patterns act similarly on several trophic levels.

When strong correlations between organismal stoichiometry and resource stoichiometry arise, they are commonly interpreted to mean that organisms are limited by the supply of energy or elements [15], [17]–[19]. The correlations observed in this study may suggest that *R. hartii* is limited by the supply of both energy (i.e. C) and nutrients (i.e. N). Energy limitation is common in fish [44], but it would be surprising if *R. hartii* was N-limited because N content in *R. hartii* is not significantly higher than N content in other freshwater taxa (Table 2), and because they feed on invertebrates which are typically rich in N. A detailed study of nutritional imbalances between *R. hartii* and their diet can help elucidate this issue.

If these correlations are causal, then they explain most, but not all, of the significant stream effect in organismal stoichiometry (Table 5), and it is likely that there are other components of this effect that we did not capture with our environmental sampling (Table 5). The influence of basal resource quality on the stoichiometry of *R. hartii* is likely mediated through invertebrate stoichiometry, because invertebrates are the dominant diet of *R. hartii*. We did not measure invertebrate stoichiometry in our study, but convincing evidence from other streams suggests that invertebrate stoichiometry is correlated with basal resource stoichiometry [17], [18]. Allochthonous subsidies may also influence the stoichiometry of *R. hartii*. This species is known to consume both aquatic and terrestrial invertebrates, and it is possible that terrestrial subsidies vary between streams [34]. However, the strongest factor influencing the use of allochthonous resources will be seasonality as *R. hartii* consume mostly aquatic invertebrates in the dry season, but their preference switches to terrestrial invertebrates in the wet season [34]. It would be interesting to test whether an increased reliance on terrestrial invertebrates after the wet season decouples organismal stoichiometry from environmental variability in stream conditions.

It is also important to note that residual spatial differences in *R. hartii* may not be entirely environmental or dietary. For example, a recent study reveals the presence of different genetic lineages of *R. hartii* in Trinidad [45]. The geographic boundaries of these lineages are poorly understood, but some appear to be defined by stream or watershed boundaries [45]. A phylogenetic signature in organismal stoichiometry has been observed even within closely related insect taxa [46], and it is possible that combining phylogenetic and stoichiometric analyses can explain the remaining portion of spatial variability in the stoichiometry of *R. hartii*.

### The influence of life history phenotype, body size and stage of maturity on organismal stoichiometry

In general, we have found organismal traits to be weaker predictors of organismal stoichiometry than streams or the interaction of stream×community. Fish community composition, which is a known selective agent of life history phenotype in *R. hartii*, is only weakly correlated with organismal stoichiometry in adult fish. Adult *R. hartii* from RG sites have significantly lower %P, and significantly higher %C, C∶N and C∶P than adult *R. hartii* from HP or RO sites. The life history phenotype of *R. hartii*, including growth rates, exists on a gradient of traits from HP to RO phenotypes, with *R. hartii* from RG sites displaying intermediate growth rates and reproductive outputs [27]. If differences in life history phenotype generate strong differences in organismal stoichiometry, we would expect N∶P and C∶N to also fall on a gradient between RO and RG locations. However, only *R. hartii* from RG sites are distinct in their stoichiometry, while HP and RO are indistinguishable, even though they are extreme ends of the life history phenotype in *R. hartii*. Though significantly correlated with community composition, %P, %C, C∶P, C∶N do not vary consistently in the direction of life history tradeoffs in *R. hartii*, suggesting perhaps that it is the interaction with guppies in RG sites that causes the significant effect of community on organismal stoichiometry. A recent study suggests that guppies inhibit foraging of *R. hartii*, which indicates that it is possible that they can also modulate the ability of *R. hartii* to acquire nutrients or energy [31], [47]. This can to be tested with experiments, but we emphasize that though effect of fish community composition on organismal stoichiometry is statistically significant, it is much smaller and weaker than the effects of stream identity or to the interaction×stream with community, which can significantly modify the signature of %P, %C, C∶N or C∶P (Fig. 4).

In *R. hartii* body size is positively correlated with %P, and negatively correlated with N∶P and C∶P. This agrees with previous studies, which have shown that larger fish contain more P-rich bone than smaller fish [9]. However, relationships between body size and elemental content are not necessarily consistent in all fish taxa, and are likely to be species-specific [48]. For example, Vrede et al. [11] have identified a significant correlation between body size and %C in perch, but we do not observe a significant relationship between body size and %C or %N in *R. hartii*. In addition, previous studies have shown that stage of maturity significantly affects nutrient content in fish [28]. *R. hartii* juveniles are not significantly different in their elemental composition compared to adults, but our findings suggest that stage of maturity modifies the effects of community on elemental composition and stoichiometry. For example, the effect of community composition on %C and C∶N is only evident in adult fish, whereas the correlation between %P and community composition is evident in both juveniles and adult fish.

### Conclusions

Our study suggests that environmental variables are stronger predictors of organismal stoichiometry than organismal traits such as body size, life history phenotype or stage of maturity. However, more is known about the influence of organismal traits on organismal stoichiometry of carnivorous taxa than is known about the importance of environmental heterogeneity. To the best of our knowledge, this is also the first study to demonstrate a significant correlation between basal resource quantity or quality and the organismal stoichiometry of an insectivorous fish in the field. These findings have important consequences for ecological stoichiometry theory because they imply that elemental stoichiometry is more likely to be constrained by the environmental availability of the elements, rather than elemental investments in organismal traits, or that environmental variability in elemental availability ultimately constrains elemental investments in organismal traits. We have highlighted three potential variables that can influence organismal stoichiometry of *R. hartii*: quality of epilithon, quality of benthic organic matter, and invertebrate standing stocks. Experiments are needed to clarify mechanisms by which environmental heterogeneity influences organismal stoichiometry in higher trophic levels. Studies also need to examine if a correlation between environmental heterogeneity and organismal stoichiometry has any bearing on the strength of homeostasis in secondary consumers. The dominant paradigm for carnivorous animals is that their elemental composition is narrow and homeostatically regulated, and as such strongly buffered from environmental or dietary variability in elemental availability [49]. A variable organismal stoichiometry that is correlated with environmental variability may suggest that carnivores are more flexible in their elemental composition than previously assumed. Because a relaxation of elemental homeostasis is likely to have significant consequences for fitness, competition and growth rates [20], the strength of elemental homeostasis needs to be examined specifically in animals that not only vary considerably in their stoichiometry, but those whose stoichiometry is strongly correlated with environmental heterogeneity in elemental availability.

## Supporting Information

Appendix S1 **Sampling of environmental variables and basal resource quality.**
(DOC)

Table S1 **Average stoichiometry of epilithon and benthic organic matter (BOM) collected from each site.**
(DOC)

Table S2 **Least squares (LS) means generated by the stage of maturity×community interaction.**
(DOCX)
