# Supplementary material for: Environmental and Organismal Predictors of Intraspecific Variation in the Stoichiometry of a Neotropical Freshwater Fish
Source: PLoS One. 2012 Mar 6;7(3):e32713. doi: 10.1371/journal.pone.0032713 (PMC3295771; doi:10.1371/journal.pone.0032713)
Supplement: Appendix S1 — Sampling of environmental variables and basal resource quality. (DOC) [file pone.0032713.s001.doc]

*Appendix S1. Sampling of environmental variables and basal resource quality*

At each site, fish and environmental variables were sampled from three representative pools and riffles. Environmental variables included stream discharge, epilithon standing stocks and quality, light availability, nutrient concentrations, and invertebrate biomass. At each site, 3 samples of filtered stream water were collected for dissolved nutrient analysis. Dissolved phosphorus was determined as soluble reactive phosphorus (SRP) using the molybdate method , and measured on a Shimadzu spectrophotometer (Shimadzu Scientific Instruments, Columbia, MD, USA). Dissolved ammonium was determined using the fluorometric method , and measured on an Aquafluor handheld fluorometer (Turner Designs, Sunnyvale, CA, USA), equipped with a UV filter. Nitrate concentrations were determined using ion chromatography, and measured on a Dionex ICS-90 Ion Chromatography System with a Dionex Automated Sampler and Chromeleon software (Dionex Corporation, Sunnyvale, CA, USA). Total dissolved inorganic nitrogen (DIN) was estimated as the sum of nitrogen from ammonium and nitrate.

Epilithon standing stocks were sampled by scraping 10 medium-sized rocks from five pools and five riffles at each site. A 3-ml subsample of the scraped slurry was immediately filtered on to a 25 mm pre-combusted glass fiber filter (Gelman GFF at 450^o^C for 2 hours, then extracted in 90% ethanol for 24 hours in the dark, after which the concentration of pigments (chlorophyll a) was measured fluorometrically (on an Aquaflour handheld fluorometer, Turner Designs, Sunnyvale, CA, USA) and corrected for rock surface area . A subsample of the slurry was dried and analyzed for stoichiometry as described in the article text. Spinach leaves were used as an internal standard for particulate %P analysis.

Fine benthic organic matter (BOM) standing stocks were measured from three pools and three riffles at each site. A known area of substrate was agitated and the suspended material sub-sampled was then dried at 50^o^C. Material greater than1000 mm was removed from the slurry. The ash-free dry mass of FBOM (g dry mass per m^2^) was estimated by weighing the dried material before and after ashing at 450^o^C for 2 hours. A subsample of the slurry was dried and analyzed for stoichiometry as described previously.

Invertebrate biomass was collected from three pools and three riffles within each site using a Hess sampler (0.25 m diameter, 250 μm mesh). Invertebrates were identified to the species level when possible. Total biomass (mg dry mass per m^2^) was estimated by applying length-mass linear regressions to species counts .

Light availability was determined as percent open canopy at each transact was measured using a hemispherical densiometer . Whenever possible stream discharge (in L per S) was measured using the midsection method . All environmental variables are summarized in Table 1. Due to logistical constraints, we were unable to measure a few of the environmental variables in all sites.

There were no significant differences in temperature (average ~ 24^o^C), dissolved oxygen (average ~ 8 mg per L) or pH (average 7.9) between sites (data not shown, but see ). All environmental sampling was carried out concurrently with fish sampling, except for epilithon, which was sampled in both July 2007-2008 and in Feb 2009. Evidence suggests though epilithon C: nutrient ratios were slightly higher in Feb 2009 than they were during the June-July 2008, but that differences between sites were maintained . We averaged values from both surveys together to account for these differences. Epilithon data reported here are also part of a much larger study on epilithon dynamics in Trinidadian streams .

References

1. Parsons TR, Maita Y, Lalli CM (1984) A manual of chemical and biological methods for seawater analysis. Oxford Oxfordshire ; New York: Pergamon Press. xiv, 173 p. p.

2. Holmes RM, Aminot A, Kerouel R, Hooker BA, Peterson BJ (1999) A simple and precise method for measuring ammonium in marine and freshwater ecosystems. Canadian Journal of Fisheries and Aquatic Sciences 56: 1801-1808.

3. Taylor BW, Keep CF, Hall RO, Koch BJ, Tronstad LM, et al. (2007) Improving the fluorometric ammonium method: matrix effects, background fluorescence, and standard additions. Journal of the North American Benthological Society 26: 167-177.

4. Benke AC, Huryn AD, Smock LA, Wallace JB (1999) Length-mass relationships for freshwater macroinvertebrates in North America with particular reference to the southeastern United States. Journal of the North American Benthological Society 18: 308-343.

5. Lemmon PE (1956) A spherical densiometer for estimating forest overstory density. Forest Science 2: 314-320.

6. Gore JA (2006) Discharge measurements and streamflow analysis. In: Hauer FR, Lamberti GA, editors. Methods in Stream Ecology Burlington, MA: Elsevier. pp. 51-78.

7. Walsh MR, Reznick DN (2008) Interactions between the direct and indirect effects of predators determine life history evolution in a killifish. Proceedings of the National Academy of Sciences of the United States of America 105: 594-599.

8. Kohler TJ (2010) Influence of Canopy Cover, Nutrients, and Season on Stoichiometric Variation of Epilithon in Neotropical Streams. Lincoln, NB, USA: University of Nebraska - Lincoln.
