# Supplementary material for: Environmental and Organismal Predictors of Intraspecific Variation in the Stoichiometry of a Neotropical Freshwater Fish
Source: PLoS One. 2012 Mar 6;7(3):e32713. doi: 10.1371/journal.pone.0032713 (PMC3295771; doi:10.1371/journal.pone.0032713)
Supplement: Table S1 — Average stoichiometry of epilithon and benthic organic matter (BOM) collected from each site. (DOC) [file pone.0032713.s002.doc]

Table S1. Average stoichiometry of epilithon and benthic organic matter (BOM) collected from each site. N/A indicates sites where logistical constraints prevented sample collection

| **Compartment** | **Site** | **Community** | **N:P** | **C:P** | **C:N** |
| --- | --- | --- | --- | --- | --- |
| BOM | Arima | HP | 16.9 | 260.10 | 15.85 |
|  | Arima | RG | 25.0 | 389.24 | 15.96 |
|  | Arima | RO | 19.5 | 346.92 | 18.30 |
|  | Aripo | HP | 20.7 | N/A | 9.58 |
|  | Aripo | RG | 39.3 | 567.86 | 14.00 |
|  | Aripo | RO | 201.0 | 878.44 | 4.57 |
|  | Guanapo | HP | 41.0 | 503.11 | 12.60 |
|  | Guanapo | RG | 39.2 | N/A | N/A |
|  | Guanapo | RO | 36.0 | N/A | N/A |
|  | Marianne | HP | 100.9 | 183.11 | 4.82 |
|  | Marianne | RG | 25.4 | 175.88 | 4.98 |
|  | Marianne | RO | 24.9 | 461.24 | 4.71 |
|  | Quare | HP | 20.1 | N/A | N/A |
|  | Quare | RG | 20.9 | 450.17 | 18.03 |
|  | Quare | RO | 23.8 | 557.73 | 23.81 |
|  | Turure | HP | 16.9 | 371.81 | 19.12 |
|  | Turure | RG | 25.0 | 525.69 | 25.67 |
|  | Turure | RO | 19.5 | 377.07 | 16.04 |
|  |  |  |  |  |  |
| Epilithon | Arima | HP | 17.7 | 160.8 | 9.1 |
|  | Arima | RG | 26.7 | 287.1 | 10.1 |
|  | Arima | RO | 23.1 | 219.5 | 9.7 |
|  | Aripo | HP | 25.7 | 181.4 | 8.8 |
|  | Aripo | RG | 30.6 | 231.9 | 8.2 |
|  | Aripo | RO | 62.1 | 436.8 | 8.9 |
|  | Guanapo | HP | 18.2 | 161.5 | 9.0 |
|  | Guanapo | RG | 18.6 | 184.1 | 7.9 |
|  | Guanapo | RO | 17.6 | 200.5 | 11.0 |
|  | Marianne | HP | 29.1 | 241.4 | 7.6 |
|  | Marianne | RG | 58.4 | 520.6 | 8.2 |
|  | Marianne | RO | 78.3 | 603.6 | 7.2 |
|  | Quare | HP | 44.7 | 470.2 | 12.0 |
|  | Quare | RG | 31.3 | 373.8 | 12.1 |
|  | Quare | RO | 18.3 | 202.2 | 11.3 |
|  | Turure | HP | 25.3 | 299.4 | 12.2 |
|  | Turure | RG | 27.9 | 363.1 | 14.1 |
|  | Turure | RO | 23.2 | 237.7 | 10.6 |
