# Supplementary material for: Environmental and Organismal Predictors of Intraspecific Variation in the Stoichiometry of a Neotropical Freshwater Fish
Source: PLoS One. 2012 Mar 6;7(3):e32713. doi: 10.1371/journal.pone.0032713 (PMC3295771; doi:10.1371/journal.pone.0032713)
Supplement: Table S2 — Least squares (LS) means generated by the stage of maturity×community interaction. (DOCX) [file pone.0032713.s003.docx]

Table S2. Least square (LS) means generated by the stage of maturity x community interaction on a general linear model performed on all individuals from the Arima, Quare and Turure. Different superscript letters denote significant differences detected by Tukey’s HSD test at alpha = 0.05.

| Variable | Community | Stage | LS mean | Standard error |
| --- | --- | --- | --- | --- |
| %C | RO | Adult | 41.0^B^ | 0.4 |
|  | RG | Adult | 43.0^A^ | 0.3 |
|  | HP | Adult | 41.0^B^ | 0.4 |
|  |  |  |  |  |
|  | RO | Juvenile | 40.7^B^ | 0.7 |
|  | RG | Juvenile | 40.7^B^ | 0.5 |
|  | HP | Juvenile | 40.7^B^ | 0.6 |
|  |  |  |  |  |
| C:P | RO | Adult | 33.3^B^ | 1.3 |
|  | RG | Adult | 36.4^A^ | 1.7 |
|  | HP | Adult | 32.3^B^ | 1.1 |
|  |  |  |  |  |
|  | RO | Juvenile | 34.9^B^ | 1.3 |
|  | RG | Juvenile | 29.9^B^ | 1.9 |
|  | HP | Juvenile | 30.0^B^ | 1.9 |
|  |  |  |  |  |
| C:N | RO | Adult | 4.4^B^ | 0.04 |
|  | RG | Adult | 4.5^A^ | 0.04 |
|  | HP | Adult | 4.4^B^ | 0.04 |
|  |  |  |  |  |
|  | RO | Juvenile | 4.4^A^ | 0.1 |
|  | RG | Juvenile | 4.2^AB^ | 0.1 |
|  | HP | Juvenile | 4.4^A^ | 0.1 |
